# Promising Pathways for Regional Disease Surveillance Networks

**DOI:** 10.3402/ehtj.v6i0.19961

**Published:** 2013-01-25

**Authors:** Melinda Moore, Katherine C. Bond, Louise Gresham, Mark Rweyemamu, A. Mushtaque R. Chowdhury, Silvia Bino

**Affiliations:** 1 RAND Corporation, Washington, D.C., United States; 2 Former Associate Director for Health, Rockefeller Foundation Southeast Asia and Africa Regional Offices, United States; 3 Fondation Merieux USA, Inc., United States; 4 SACIDS at Sokoine University of Agriculture, Morogoro, Tanzania; 5 Rockefeller Foundation, Thailand; 6 Regional Development Center of Communicable Diseases Surveillance and Control, Institute of Public Health, Tirana, Albania

## Introduction

The globalization of trade and travel has led to the globalization of communicable diseases and, in turn, increased need for globalization of solutions to fight them. The self-organized regional disease surveillance networks described in this special issue of *Emerging Health Threats* are one such solution. They reflect the vision, commitment and leadership of country health leaders and their development partners (1–4). The networks described here are significantly different from and complementary to regional surveillance systems of the World Health Organization (WHO), World Organization for Animal Health (OIE) and Food and Agriculture Organization (FAO) (5, 6). They are more literally “sub-regional” – organized by a smaller number of countries and built on a foundation of trust, cooperation, and mutual public health interests; they connect “bottom-up” local, national, and neighboring trans-national surveillance to “top-down” global and larger regional systems through “horizontal” cooperation across borders, disciplines, and sectors. These networks have demonstrated their value, judging from formal evaluations (7–11) and based on networks’ own descriptions of joint investigations of priority diseases and other activities that ranged from human and system capacity building to pandemic preparedness and regional support to a member country following a major natural disaster (12–17). But challenges remain. The networks can continue to learn and improve, building on a strong foundation of mutual trust, informal and formal communications, and regional cooperation. They can better inform and intersect with efforts addressing other regional and global public health challenges, such as natural disasters, antimicrobial resistance, and product safety.

This paper describes five promising pathways for these networks, based on lessons derived from their experiences to date and a shared desire to lean confidently into the future. The strategic objectives of Connecting Organizations for Regional Disease Surveillance (CORDS) – build sustainable networks, improve capacity, advance One Health, and promote innovation – fully support the five promising pathways (18). Moreover, CORDS provides a means for regional networks to share experiences and work together as they proceed down these pathways toward improvement. CORDS played a vital role in bringing the regional networks together and will catalyze future cooperation (18, 19).

## Five Promising Pathways

The challenges of emerging infectious diseases are even more acute in these times of global austerity, when resources are especially limited and must be used efficiently and creatively. To address these challenges, we describe a new “Promising Pathways Model” with five promising pathways that are encompassed within three key concepts: *Accountability* (pathway 1) is enhanced through the use of standardized international monitoring frameworks. *Cooperation* across disciplines/sectors (pathway 2) and borders (pathway 3) enables rapid and coherent public health responses. *Innovation* in technologies (pathway 4) and regional network business models (pathway 5) provides new approaches to public health surveillance and network sustainability.

### Accountability: Use global frameworks to guide network capability monitoring

The WHO International Health Regulations (IHR) modernized the global approach to disease surveillance and early warning and articulated the responsibilities of countries to prepare for and respond to public health emergencies of international concern (20). The IHR defines core capacities related to surveillance, response, preparedness, risk communication, human resources, laboratory and coordination. The OIE Terrestrial Animal Health Code complements this on the animal side (21). Interestingly, WHO recommends that IHR core *capacities* be measured according to *capability* level (22). The distinction is important, as capabilities-based planning has become international best practice for all types of emergencies–natural disasters, pandemics, accidents and intentional threats (23). Typical measures of capacity include number of persons trained, laboratories built and equipped, and computers provided; measures of capability reflect actual performance. The four-point WHO scoring scale reflects capacities (levels 0, 1) that escalate to capabilities (levels 2, 3). The OIE capability monitoring system is embodied in the Performance of Veterinary Services (PVS), which operates through a four-stage Pathway that includes evaluation, gap analysis, capacity building and maintenance (24). Both the IHR and PVS provide monitoring tools for measuring country progress that CORDS networks could use to guide their own monitoring and accountability efforts. Accountability ensures that regional surveillance is effective, efficient, consistent with international standards, responsive to local needs, and outcome-oriented.

### Cooperation across disciplines/sectors: Integrate trans-disciplinary and trans-sector efforts to improve health security and human security

The IHR 2005 galvanized the connection between health and security and the concept of “health security” (25–29). Despite differing interpretations of what this means, both health security and the related “human security,” which focuses on the welfare of common people, are pertinent to all countries (30–32) and hence to regional disease surveillance networks. Cooperation across multiple sectors – health, agriculture, finance, border security, customs and others – enhances situational awareness and response. With health concerns increasingly recognized within national security agendas (28, 30, 31, 33, 34), regional networks can contribute to improving both health security and human security by cooperating not just across borders, but also across sectors. Cooperation between the human and animal health sectors is especially important because most infectious diseases that have emerged in recent decades are of animal origin, with their emergence being linked to trade, economic, and political interests (28, 35, 36). As described throughout this issue, CORDS networks are adopting the One Health paradigm in various ways and to varying extents (e.g., see Ref 16 in this supplement for a more detailed discussion of One Health).

### Cooperation across borders: Develop coherent regional policy for rapid response and economies of scale

As exemplified by the regional disease surveillance networks described in this issue, the cross-border collaborative activities of such networks complement those of WHO, OIE and FAO for disease detection, outbreak investigation and response (37–39). These networks can also cooperate in regional procurement, such as bulk purchase of equipment or vaccines, and in developing common protocols for laboratory testing (14, 15, 40) or disease reporting.

### Innovation in technology: Capitalize on new and under-utilized technologies for data generation, analysis and action

Mobile phone, social media, geospatial, and other electronic tools enable broader coverage and faster disease surveillance, prevention, and control. CORDS networks are capitalizing on these and other technology innovations to improve both national public health systems of their respective countries and network cooperation across countries. For example, SACIDS is piloting a One Health-based mobile telephone disease surveillance tool. The phones are equipped with data capture and epidemiological analytical software (17, 41). Some of these technologies can also enable the public to play a more active role in public health surveillance. For example, MBDS has a core strategy for community-based surveillance, which calls for reporting of unusual events by community members (12, 42). Harnessing innovations for surveillance is effective only if the captured information is actually used. Studies in various parts of the world have demonstrated deficiencies in actually using surveillance for management and action (43–46). Regional networks are well suited to ensure that surveillance data are not only shared from their own local to national levels, but also across sectors and borders.

### Innovation in disease surveillance network business models: Create new, flexible models to attract resources and foster sustainability

CORDS regional disease surveillance networks exemplify owner-driven, donor-supported agendas, which are significantly different from the typically donor-driven agendas of yesteryear. Each network has established its own governance structure, and network member countries participate actively in setting priorities for their cooperation. For example, MBDS development partners have supported network efforts based on the countries’ own collective master plan of action (47), which also helps to improve donor coordination; MECIDS countries decided to cooperate initially around surveillance for foodborne pathogens (40); and SACIDS institutions decided to focus on zoonotic diseases (48).

Sustainability of the networks will depend on multiple factors: owner-driven agendas, the enduring trust among network members and their commitment to remain engaged even through times of political turmoil and resource challenges, fruitful partnerships to support network priorities, alignment with international standards, and a culture of accountability (5). While the networks featured here have built strong platforms for cooperation, sustainable cooperation requires conscious action. The networks are evolving as centers of excellence for regional cooperation. As such, they are well positioned to seek research grants, technology investments by the private sector, and support from other sources interested in utilizing these established regional population laboratories as models for further development and study. The establishment of the MBDS Foundation as a permanent entity agreed upon by the six countries is one approach to this. Publication of regional network activity (4, 12–17, 48–51) further increases their visibility and credibility, enhances opportunities to attract further investment, and thereby strengthens their prospects for sustainability. A shared interest in the health security of each country and global health diplomacy across countries are further foundations for regional network sustainability into the future.

## The Role of CORDS

CORDS was established to link existing regional disease surveillance networks and foster new cross-border and cross-sectoral learning and innovation (18, 19, 52). As such, CORDS is strategically poised to catalyze progress along the five promising pathways described here and to help these surveillance networks harness their full potential to improve global health.
